# COVID infection in 4 steps: Thermodynamic considerations reveal how viral mucosal diffusion, target receptor affinity and furin cleavage act in concert to drive the nature and degree of infection in human COVID-19 disease

**DOI:** 10.1016/j.heliyon.2023.e17174

**Published:** 2023-06-12

**Authors:** Marko Popovic, Jennifer H. Martin, Richard J. Head

**Affiliations:** a Institute of Chemistry, Technology and Metallurgy, University of Belgrade, Njegoševa 12, 11000 Belgrade, Serbia; b Centre for Drug Repurposing and Medicines Research, University of Newcastle and Hunter Medical Research Institute, Newcastle 2305, Australia; c Drug Discovery and Development, Clinical and Health Sciences, University of South Australia, Adelaide, South Australia, Australia

**Keywords:** Viral diffusion, Pharmacology, Affinity constant, Thermodynamics, Human disease, SARS-CoV2, COVID-19, SARS-CoV, ACE2

## Abstract

We have developed a mechanistic model of SARS-CoV-2 and SARS-CoV infection, exploring the relationship between the viral diffusion in the mucosa and viral affinity for the angiotensin converting enzyme 2 (ACE2) target. Utilising the structural similarity of SARS-CoV and SARS-CoV-2 and a shared viral target receptor (ACE2), but a dramatic difference in upper or lower respiratory tract infectivity, we were able to generate insights into the linkage of mucosal diffusion and target receptor affinity in determining the pathophysiological pathways of these two viruses.

Our analysis reveals that for SARS-CoV-2 the higher affinity of ACE2 binding, the faster and more complete the mucosal diffusion in its transport from the upper airway to the region of the ACE2 target on the epithelium. This diffusional process is essential for the presentation of this virus to the furin catalysed highly efficient entry and infection process in the upper respiratory tract epithelial cells. A failure of SARS-CoV to follow this path is associated with lower respiratory tract infection and decreased infectivity. Thus, our analysis supports the view that through tropism SARS-CoV-2 has evolved a highly efficient membrane entry process that can act in concert with a high binding affinity of this virus and its variants for its ACE2 which in turn promotes enhanced movement of the virus from airway to epithelium. In this way ongoing mutations yielding higher affinities of SARS-CoV-2 for the ACE2 target becomes the basis for higher upper respiratory tract infectivity and greater viral spread. It is concluded that SARS-CoV-2 is constrained in the extent of its activities by the fundamental laws of physics and thermodynamics. Laws that describe diffusion and molecular binding. Moreover it can be speculated that the very earliest contact of this virus with the human mucosa defines the pathogenesis of this infection.

## 1 Introduction

Thomas Huxley: “Nothing can be more incorrect than the assumption one sometimes meets with, that physics has one method, chemistry another, and biology a third.” (Aphorisms and Reflections from the Works of T. H. Huxley.)

Both the virus and its human host represent growing open thermodynamic systems [[1], [2], [3]]. They interact biologically, chemically and thermodynamically [4]. There are four steps of virus-host interaction: (1) inhalation of aerosol [5], (2) diffusion of viral particles through mucosal layer, (3) antigen-receptor binding and viral entry through host cell membrane [[6], [7], [8]], and (4) viral multiplication inside the host cytoplasm and self-assembly [9]. Each process is driven by a physical driving force [2,10,11]. An analysis of the drivers of SARS-CoV2 infection and its pathogenicity has shown that COVID-19 disease is a consequence of a lethal viral simplicity that transverses human health, biology, physics and chemistry. Understanding how the biological impact of SARS-CoV2 is both constrained and facilitated by the fundamental laws of physics, chemistry and thermodynamics is much needed [2,3,10,[12], [13], [14]]. In order to achieve this, a mechanistic physicochemical model of SARS-CoV2 infection is necessary [[15], [16], [17], [18]]. Thermodynamic properties of SARS-CoV, MERS-CoV and all major SARS-CoV2 variants are available in the literature [12,[19], [20], [21], [22], [23], [24], [25], [26], [27], [28], [29], [30], [31], [32]].

At its essence SARS-CoV2 infection is driven by the chemistry of self-replication at scale, catalysed by our own cellular processes in a fashion favourable to the rigors of thermodynamics in permitting this self-assembly [32]. A cascading self-replication within a single cell, a tissue, an organ, a human, and an entire population [14,19,33,34]. This self-replication is fuelled by favourable Gibbs free energy considerations made possible by a viral tropism that makes angiotensin converting enzyme 2 (ACE2) a high affinity target for SARS-CoV2 [19,26]. The Gibbs energy of binding drives the infectivity of SARS-CoV2 [20]. Mutated variants of SARS-CoV2 not only have different Gibbs energies of binding but through mutation display more negative Gibbs energy values [19,21].

Globally, as of April 12, 2023, there have been 762,791,152 confirmed cases of COVID-19, with 6,897,025 deaths, reported to WHO [35]. A total of 13,340,275,493 vaccine doses have been administered [35]. COVID-19 vaccines can be sorted into four main types: RNA-based (BNT16b2 by Pfizer/BioNtech; mRNA-1273 by Moderna; CVnCoV by CureVac), viral vector (AZD1222 ChAdOx1 nCoV-19 vaccine by AstraZeneca/University of Oxford; Ad26.COV2.S by Johnson & Johnson; Gam-COVID-Vax Sputnik V by Gamaleya Research Institute; Convidecia™ Ad5-nCoV by CanSino), Protein-based (NVX-CoV2373 by Novavax; EpiVacCorona by VECTOR; ZF2001 by Institute of Microbiology of the Chinese Academy of Sciences and Anhui Zhifei Longcom Biopharmaceutical; CIGB-66 Abdala by Center for Genetic Engineering and Biotechnology CIGB) and inactivated virus (CoronaVac by Sinovac Biotech; BBIBP-COrV by Sinopharm/Beijing Institute of Biological Products; Wuhan by Sinopharm/Chinese Academy of Science; Covaxin by Bharat Biotech) [36]. Moreover, therapeutic strategies for COVID-19 have been discussed, such as extracellular vesicle-based therapy [37]. The contribution of extracellular vesicles to research on COVID-19 pathogenesis with an analysis of clinical observations is given in Ref. [37]. There is a need to expand the knowledge base regarding the properties SARS-CoV2 generated from molecular biology, immunology and virology by molecular biologists, to additionally incorporate mechanistic models that include biothermodynamics.

Previously we highlighted the significance of the shedding of SARS-CoV2 from the epithelium of nasopharyngeal tissues from an infected individual to its subsequent attachment to the ACE2 viral target on the nasal epithelial cells of a new host [14]. As indicated by Li et al. [38], virus enters the upper respiratory tract after virus containing droplets are deposited on the mucus layer on the airway surface. This initial interaction with the mucosal surface and the activities immediately following this contact are pivotal in COVID-19 disease. However, what occurs during this passage toward the target cells is often ignored during viral load calculations.

We believe this paucity in knowledge on the events associated with the first contact of SARS-CoV2 with a human represents a key unmet need in understanding the earliest events in infection driven by SARS-CoV2. Accordingly, we have employed the fundamental thermodynamic principles underpinning diffusion and linkage to receptor binding to provide a better understanding of the very inception of COVID-19 disease in the human built upon of two of the fundamental laws in science (Fick's law of diffusion and Gibbs free energy of binding) as well as Le Châtelier's principle to describe the earliest event in COVID-19 disease.

## 2 Methods

The methods employed involved the derivation and application of equations and modelling based on diffusional flux to target receptors and its interplay with dissociation constants associated with receptor binding. The equation derivations, the subsequent mechanistic models and the relationships generated are documented in the Supplementary Section (9.1 and 9.2).

### 2.1 Data sources

Dissociation constants for SARS-CoV and Hu-1 variant of SARS-CoV-2 were taken from Walls et al. [39]. The dissociation constant, *K*, of SARS-CoV is 5.0 nM, while *K* of the Hu-1 variant of SARS-CoV-2 is 1.2 nM, at 30 °C [39].

### 2.2 Mechanistic model of virus absorption, diffusion and binding

A mechanistic model is developed of the initial entry of viral particles into the organism, which is shown in Fig. 2. The virus particles initially enter the lumen of the respiratory pathways with air. The virus particles dispersed in air within the lumen are designated as the initial state N. Once inside the respiratory pathways, the virus particles come into contact with the mucus surface. This is where the first process begins, which represents an absorption of virus particles from the air into the mucus surface layer. Some of the particles enter the mucus surface, which is designated as state A. Once absorbed in the mucus surface, the virus particles begin to diffuse to the pericilliary layer at the surface of the host tissue (bottom of the mucus). This is designated as state C and the second process is diffusion from state B to C. Finally, the virus particles in the pericilliary layer bind to host cell ACE2 receptors. This is designated as state D. Thus, the virus particles can be in four states: in the air (N), in the mucus surface layer (A), unbound in the pericilliary layer (B) and bound to the host cell receptors (C).

The states described above are connected by processes. The first process (N - > A) is the absorption of virus particles from the nonpolar air into the polar mucus surface. The second process (A - > B) is diffusion of virus particles from the mucus surface to the pericilliary layer. The third process (B - > C) is binding of virus particles to the ACE2 receptors on the host cell surface. Thus we have an absorption process, a diffusion process and a chemical reaction. More about the model can be found in the Supplementary Section (9.1).

### 2.3 Biophysical analysis of the absorption, diffusion and binding model

The proposed mechanistic model of absorption, diffusion and binding consists of four states (N, A, B and C), which are connected by three processes. The first process (N - > A) is absorption of virus particles from the air into the mucus surface. This process is described by the partition coefficient, *f*,

(1) $$f=\frac{n}{a}$$

where *n* is the concentration of virus particles in the air and *a* the concentration of virus particles in the mucus surface layer [40]. The second process (A - > B) is diffusion of virus particles from the mucus surface layer to the pericilliary layer. This process is described through Fick's law

(2) $$J=-D\frac{a-b}{x}$$

where *J* is the flux of virus particles from the mucus surface to the pericilliary layer, *D* the diffusion coefficient, *b* the concentration of virus particles at the pericilliary layer and *x* the thickness of the mucus layer [41,42]. The third process (B - > C) is binding of virus particles to the host cell ACE2 receptors. It is described by the dissociation constant, *K*,

(3) $$K=\frac{b∙r}{c}$$

where *r* is the concentration of the ACE2 receptors in the host tissue and *c* the concentration of virus particles bound to the host cell receptors. Finally, there is also the law of conservation of matter, which states that the initial number of virus particles taken into the organism is equal to the sum of virus particles in the four states

(4) m=a+b+c+n

where m is the total concentration of viruses taken into the organism [19,43]. Therefore, the development of the model begins from equations (1), (2), (3), (4).

The system of equations (1), (2), (3), (4) presented above was solved to find how the concentrations of virus particles in the four states (*n*, *a*, *b* and *c*) change with time. The solution procedure is presented in detail in the Supplementary Section (9.2). The final solutions of the equations are

(5) $$\frac{a}{m}=e^{-t/\tau }+\frac{K}{r}$$

(6) $$\frac{b}{m}=\frac{K}{r}\left(1-e^{-t/\tau }\right)$$

(7) $$\frac{c}{m}=1-e^{-t/\tau }$$

(8) $$\frac{n}{m}=f\left(e^{-t/\tau }+\frac{K}{r}\right)$$

where *τ* is the time constant, specific for the system, which is defined by the diffusion coefficient, *D*, and mucus thickness, *x*,

(9) $$\tau =\frac{D}{x^{2}}$$

### 2.4 Gibbs energy of binding

One of the main parameters in the model above is the dissociation constant, *K*, which describes the process of binding to host cell ACE2 receptors. The dissociation constant is based on a molecular-level parameter: Gibbs energy of binding, Δ_*B*_*G⁰*, and given by the equation

(10) $$K=\exp\left(+\frac{\Delta_{B}G^{0}}{RT}\right)$$

where *R* is the universal gas constant and *T* temperature (the plus sign instead of minus was introduced since binding and dissociation are opposite processes) [19]. Thus, *K* is proportional to the exponent of Δ_*B*_*G⁰*. This means that Gibbs energy of binding is, along with viral diffusion properties *τ* and *D*, one of the main parameters that determines the outcome of the early infection process, according to equations (5), (6), (7), (8), (9).

The dissociation constant is proportional to the exponent in Gibbs energy of binding. This means that even small changes in Gibbs energy of binding lead to large changes in the dissociation constant *K*. This is in agreement with the finding that the differences in Gibbs energies of binding between the different variants of SARS-CoV-2 lead to large differences in infectivity [44].

Gibbs energy of binding also has a great influence on the number of virus particles that enter the lower respiratory pathways. Virus particles enter the organism with air and travel along the respiratory pathways: through the nasal cavity, oral cavity, larynx, trachea etc. to the lower respiratory pathways. Thus, some time needs to pass before they enter the lower respiratory pathways. For large values of time (*t* → ∞), equation (8) simplifies into

(11) $$\frac{n}{m}=\frac{f}{r}\;K$$

This means that the concentration of virus particles in the air that reaches the lower respiratory pathways is proportional to the dissociation constant *K*. This means that Gibbs energy of binding determines the number of virus particles that reaches the lower respiratory pathways. The stronger the binding, the more negative the Δ_*B*_*G⁰*. The more negative Δ_*B*_*G⁰*, the lower the *K* value according to equation (10) and the less virus particles will reach the lower respiratory pathways, according to equation (11). More about this can be found in Supplementary Section (9.3).

## 3 Results

**Fig. 1:**
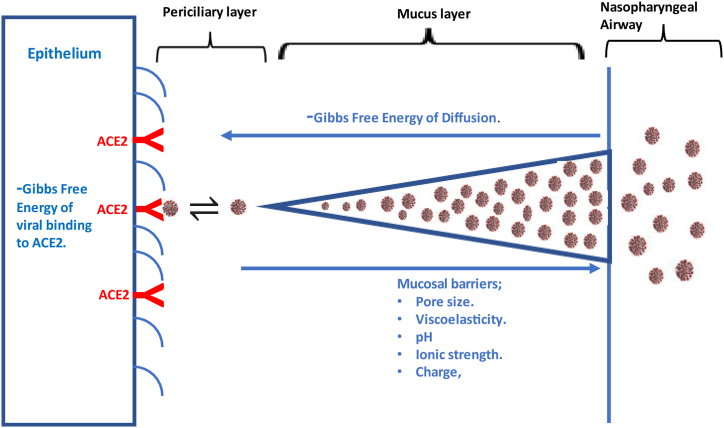
**A schematic representation of the virus journey across to the epithelium.** Legend. represents the passage of the virus (SARS-CoV or SARS-CoV2) from the upper respiratory tract airway across the mucosal barriers to viral receptor target ACE2 on the airway epithelium. The representation reflects events immediately after the inhalation of virus containing droplets or aerosols and prior to viral entry into the epithelium. The thickness of the arrow is illustrative of the diffusion gradient of the virus across the mucosal layers determined by the interplay of the molecular diffusion and the sum of the individual opposing contributions within the mucosal barrier. Of note is the equilibrium established between receptor bound and non-bound virus in the periciliary layer adjacent to the epithelial cell bound ACE2 target. The importance of the mucosal barrier and the physicochemical properties of pore size, visco-elasticity, pH, ionic strength, and charge have been illustrated well for drug delivery [45].

The results are presented as a series of responses to key questions associated with the passage of viruses (with particular emphasis on SARS-CoV and SARS-CoV2) from the upper respiratory tract mucosal-airway interface and the subsequent viral passage across the mucosal layers to the region of the viral receptor target (ACE2) on the epithelium. These features are illustrated in Fig. 1.

**Fig. 2:**
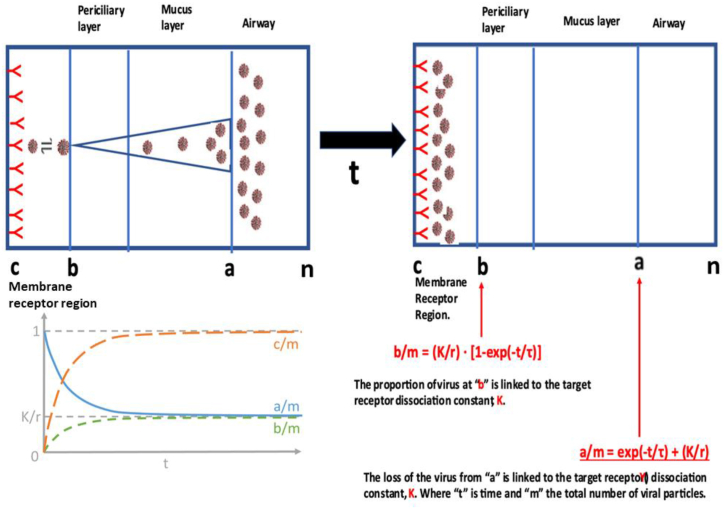
**Receptor affinity and mucosal diffusion.** Legend. Shown is a schematic and linked graphical representation of the relationship between viral receptor affinity and mucosal diffusional flux. Note the free virus particles at the mucus surface, “a”, decreases exponentially with time and virus bound to the receptors increases with time and ultimately accounts for the bulk of the migrated virus. **a** = concentration of free virus at the mucus surface. **b** = concentration of free virus adjacent to epithelial cell membrane surface. **c** = concentration of virus particles bound to epithelial cell ACE2 receptors. **m** = total concentration of virus initially arriving in the airway. **n** = concentration of free virus particles in the airway at time t after arrival of the air. The model illustrates the passage of the virus from when it enters the mucosal barrier (upper left panel) with a concentration of and at (“a”), diffuses with time (“t”) through the mucosal layer to the region of the epithelial membrane (upper right panel) with a concentration of and at (“b”) and in equilibrium can bind to the ACE2 receptor with a concentration of and at (“c”).

Highlighted in Fig. 1 is the concept that virus concentration across the mucosal layers is determined by the interplay of diffusional flux, the affinity of the virus for the ACE2 receptor target and the opposing influence of the mucosal barriers. The intersection of diffusion and receptor binding in this context is defined by the thermodynamic properties described by Fick's law of diffusion and Gibbs energy of antigen-receptor binding. For convenience the following discussion utilises diffusional flux and receptor dissociation and affinity constants as derivatives of the thermodynamic measures.

### 3.1 Is there a relationship between viral receptor affinity and viral mucosal diffusional flux?

From the integration (see) Supplement 9.2 of Fick's Law of diffusion (applied to virus concentration from high to low across a gradient), the target receptor Dissociation Constant (the measure of the associated and disassociated virus with its ACE2 target receptor) and the Law of Conservation (the total viral mass must be the same before and after the migration across the mucosal barrier) we established the following:

• A relationship exists between the affinity for a virus in binding to ACE2 target and the viral diffusion across the mucosal layer. A mechanistic biophysical model was developed to describe and explain the relationship.
• Using this mechanistic biophysical model, it is possible to follow the migration of the virus from the airway-mucosal surface, across the mucosal layers to the region of the epithelial membrane surface where it is bound to the target receptor.

These features are summarised in Fig. 2 where it can be seen that the concentration of free virus particles at the mucus surface, “a”, decreases exponentially with time with all virus particles progressing from a/m = 1, to a small constant concentration of a/m = *K/*r. In addition, the concentration of free virus particles at the membrane surface, “b”, increases very slowly: from b/m = 0 to b/m = K/r. This means that the viruses take some time to diffuse to the membrane surface where most of the virus is bound, because the dissociation constant is very small. Finally, the concentration of virus particles bound to the host membrane, “c”, increases through time: from 0 to c/m ≈ 1, meaning that almost all of the virus particles (“m”) are bound.

The most informative consideration is encapsulated in the relationship between the concentration of virus at the mucus surface (“a”) and the dissociation constant (K) namely a/m = exp(-t/τ) + (K/r). This conclusion follows from the fact that the affinity constant is equal to the reciprocal of the dissociation constant and supports the view that the degree to which the virus will be sequestered from the airway, to cross the mucosal layer to infect the underlying epithelium, is driven by the affinity of the virus for the target receptor ACE2. A similar relationship between the dissociation constant (K) and the virus concentration at the epithelial membrane surface is also evident in the relationship b/m = (K/r) ⋅ [1-exp(-t/τ)].

This analysis means it is now possible to include mucosal diffusion in relate differing pathophysiological pathways (e.g., upper versus lower respiratory tract infection) with viruses displaying differing affinities for the ACE2 receptor target.

From the Supplement section the changes in concentration of a, b, and c with time are given by.

a/m=exp(−t/τ)+(K/r)

b/m=(K/r)⋅[1−exp(−t/τ)]

c/m=1–exp(−t/τ)

n/m=f⋅[exp(−t/τ)+(K/r)]

where (*r*) is the concentration of ACE2 receptors, (*x*) is mucous thickness and *τ* is the time constant, specific for this system, defined by the diffusion coefficient (“*D*”) and mucus thickness, (*f*) is a coefficient describing partition of viruses between the mucus surface and air. Thus *τ* = *D*/*x*^2^. The assumptions include host cell receptors (*r*) being very much greater than the dissociation constant of the virus (*K*). Because the focus is solely on mucosal diffusion and receptor binding entry of the virus into the epithelium has not been considered in this analysis.

### 3.2 What is the influence of an increased viral receptor affinity of variants on the viral mucosal flux?

A key observation has been that the Gibbs energies of binding to the viral target decrease chronologically, with appearance of new variants [19]. In the previous section we described a dynamic equilibrium existing between viral passage across the mucosal layer and the affinity of binding to the ACE2 viral target. With variants this equilibrium is altered and based upon Le Chatelier's principle a new dynamic equilibrium must be established which in this setting is achieved by altering the rate of viral diffusion. As summarised in the supplement (9.2) we have used the intersect of Le Chatelier's principle and Fick's Law of diffusion to determine if the mutated strains of SARS-CoV2 displaying more negative Gibbs energy values [19] have enhanced diffusion across the mucosal barrier. The data for all variants is shown in the supplementary data where the following is evident (sections 9.3 and 9.4).

Firstly, the curve of a/m is the same for all the variants only for small t (Supplement: Figure S3) where the exponent term dominates, which describes diffusion and is the same for all the variants.

Secondly at large t, the exponent term becomes small, and the K/r term dominates and since K is different for every variant, the a/m curves are different for all the variants (Supplement: Figure S4). Thus, the more negative Gibbs energy associated with viral receptor binding the faster the virus can travel through the mucus and gain entry to the host.

### 3.3 How the comparative pathophysiology of SARS-CoV and SARS-CoV2 infection reveals the role of thermodynamically linked viral mucosal flux with target receptor affinity in defining COVID-19 disease?

SARS-CoV and SARS-CoV2 are remarkably similar viruses in physical composition [12,22,25]. They share as much as a 79.5% identity in genome sequence [46] and the envelope proteins, the spike proteins, and the nucleocapsid protein share identity and similarity percentages ranging from 76.5% to 96.4% [47]. Accordingly, it can be assumed the mucosal diffusional characteristics of SARS-CoV and SARS-CoV2 will be similar.

Despite these similarities, the pathophysiology of SARS-CoV infection is dramatically different when compared with that for SARS-CoV-2 and its more recent variants. SARS-CoV mediates lower respiratory tract infection with low infectivity and SARS-CoV2 drives upper respiratory tract infection with high infectivity and population spread. It is a consideration of viral mucosal flux driven by enhanced receptor affinity that provides a rational basis for the differences in disease outcome.

SARS-CoV-2 (and its variants) display two trophic features that dictate upper airway infectivity that involve mutations that are miniscule in comparison to the overall structure of the virus. The first is binding of SARS-CoV-2 to the ACE2 receptor with a much greater affinity than the binding than displayed by SARS-CoV. In Supplement 9 we have derived the equations for the absorption, diffusion and binding of SARS-CoV and SARS-CoV2 variants. The second trophic influence is the presence in SARS-CoV2 but not SARS-CoV of a poly basic insertion permitting furin mediated S1/S2 cleavage of the spike protein enhancing SARS-CoV2 epithelial cellular entry and its removal from the extracellular mucosal space.

**Fig. 3:**
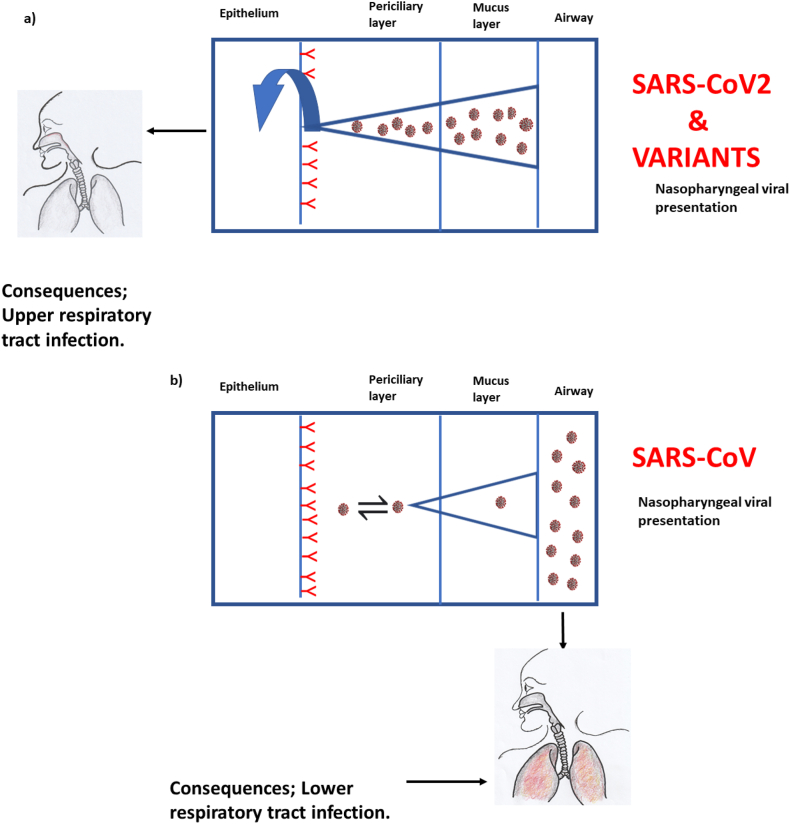
**Differences in Infection pathways with SARS-CoV and SARS-CoV2.**Fig. 3a and b legend. Shown is a schematic representation of the differences in infection pathways and consequences of SARS-CoV and SARS-CoV2 in the upper respiratory tract. (a) Epithelial Cell entry favours SARS-CoV2 over SARS-CoV by virtue of the higher binding affinity for ACE2 driving enhanced diffusion and the presence of a furin spike protein cleavage sensitivity promoting highly efficient epithelial cell entry. The combination of these two trophic processes is upper airway infection. (b) In contrast to SARS-CoV2, the lower receptor affinity of SARS-CoV results in poorer mucosal rates of diffusion and the absence of a furin substrate in the spike protein denies SARS-CoV a rapid cellular entry resulting in an equilibrium state within the periciliary area. Consequently, there is minimal upper respiratory tract infection and more non-sequestered virus available to travel to and infect the lower respiratory tract.

It can be argued that for SARS-CoV2 and its variants the enhanced binding affinity for the ACE2 receptor (see Supplement 9.4) drives a greater diffusional mediated presentation of SARS-CoV2 to a highly efficient furin mediated cellular viral entry (Fig. 3). This combined trophic influence mediated in large part by diffusion is the underlining basis for upper respiratory SARS-CoV2 infection. Thus, for SARS-CoV2 the equilibrium between the viral target receptor binding and the virus in the periciliary layer is abolished, leading to ongoing stoichiometric replacement of virus in the periciliary layer. This ensures the replenishment of SARS-CoV2 and the availability of further virus to infect additional bystander viral targets (Fig. 3).

In contrast SARS-CoV displaying less affinity for the ACE2 target and an absence of the efficient furin based cell entry all leads to a failure in inducing upper airway infection and an availability of SARS-CoV for migrate to the lower respiratory tract (Fig. 3).

The foregoing illustrates the presence a common platform of viral mucosal flux driven by enhanced receptor affinity for SARS-CoV and SARS-CoV2 that can be trophically manipulated by mutation (receptor binding and furin sensitivity) to dictate the upper or lower respiratory tract pathophysiology infection.

## 4 Discussion

Previously we have suggested that the measure of entropy that predicts the outcome of SARS-CoV2 infection is related to the thermodynamic interplay between entropy and enthalpy that governs the spontaneity of infection of a single cell [14]. In this study we have explored the critical thermodynamic contingence immediately prior to infection of nasopharyngeal epithelial cells. We have established, based on thermodynamic principals, a relationship exists between upper airway mucosal diffusion of SARS-CoV2 and its affinity for binding to its target receptor ACE2. We have provided support for the concept that the degree of viral diffusional flux is directly linked to the target receptor dissociation constant such that the driving force for viral entry is the coupled processes of binding and diffusion. From a thermodynamic perspective the combined processes are favourable.

Utilising this platform, by way of mutation driven tropism, SARS-CoV2 has evolved a fixed highly efficient membrane entry process that acts in concert with target receptor binding. As we have highlighted previously the Gibbs energies of SARS-CoV2 binding with ACE2 decrease chronologically, with appearance of new competing viral strains [19]. In this way ongoing SARS-CoV2 mutations yielding higher affinities for the ACE2 target becomes the basis for enhanced mucosal diffusion driven higher upper respiratory tract infectivity and viral spread for SARS-CoV2 and its variants.

SARS-CoV and SARS-CoV-2 represent biological, chemical and thermodynamic systems, performing the multiplication process within the host cell [32]. Virus multiplication represents a biological, chemical and thermodynamic process [48]. The driving force for all biological, chemical and thermodynamic processes in nature is Gibbs energy [2,11,49]. Gibbs energy of biosynthesis for SARS-CoV nucleocapsid is −230.3 kJ/C-mol [32], while Gibbs energy of biosynthesis of SARS-CoV-2 nucleocapsid is −222.2 kJ/C-mol [22,32]. According the biosynthesis phenomenological equation [22,44,48], this means that the multiplication rate of SARS-CoV should be greater than that of SARS-CoV-2. This leads us to the conclusion that, due to greater multiplication rate, SARS-CoV causes greater damage in the host cells. From this we can conclude that pathogenicity is greater in SARS-CoV than in SARS-CoV-2. This is in accordance with the predictions of the evolution theory that SARS-CoV-2 has evolved towards increased infectivity and decreased pathogenicity [50]. The consequence of this is that SARS-CoV has developed a more localized epidemic, with a greater fatality. However, the key is the issues of replication and pathogenicity are events subsequent to the mucosal diffusion and cell surface target receptor binding. If this combined diffusional and binding effect is large you can predict greater presentation of the virus to the epithelium in the upper airway and subsequently greater upper airway replication, shedding and infectivity.

Transmissibility is also key, thus if the combination of diffusion and receptor target binding favors upper airway passage it can be argued that transmissibility will be high. Indeed, we speculated earlier (Head et al., 2022) that the epidemiological characteristics of the COVID-19 pandemic must, in part or fully reflect the fundamental characteristics of SARS-CoV-2 target binding, assembly, and organization that occurs in a single cell, tissues or collectively in the human. Transmissibility of SARS-CoV-2 is grater due to faster binding of the virus to the ACE2 receptor and also greater number of less severe cases, which have obtained the ability to spread the virus within the population. Epidemiological measures were taken for both the SARS-CoV epidemic and SARS-CoV-2 pandemic. They have certainly played a role in suppression of the epidemic/pandemic. However, the greater spread of SARS-CoV-2 through the population and longer duration of the COVID-19 pandemic have led to a large number of mutations in the SARS-CoV-2 virus. The SARS-CoV-2 virus has mutated numerous times from the Hu-1 variant in 2019 to the newest Omicron BN.1, CH.1.1 and XBC variants [51]. Therefore, SARS-CoV-2 virus is from the evolutionary aspect one of the best characterized viruses, from the perspective of molecular biology, biochemistry and thermodynamics.

Our focus has effectively been upon the area of greatest potential vulnerability for the virus, namely in its passage from the airway across the mucosa to the binding site on the surface of the epithelium. Within this passage we argue that the key determinants are diffusion and target receptor binding acting in concert.

## 5 Conclusion

It can be concluded that the very earliest contact of this virus with the human mucosa defines the subsequent pathogenesis of this infection and reflects an ideal site for intervention. Intervention at time when the virus through tropism is at its most powerful in setting the agenda for this disease but at a time when it is paradoxically at its most vulnerable.

A mechanistic biophysical model was developed, describing virus-host interactions of SARS-CoV-2 and SARS-CoV. The model analyses viral absorption into the mucus, diffusion through the mucus, and binding to host cell receptors. The analysis was made based on thermodynamic properties – dissociation constants and Gibbs energies of binding. It shows that due to stronger binding to host cell receptors (lower dissociation constant and more negative Gibbs energy of binding), SARS-CoV-2 binds to receptors soon after entry into the organism, in the upper respiratory pathways. SARS-CoV has a weaker affinity for ACE2 receptors, due to which it binds in the lower respiratory pathways.

We suggest that SARS-CoV-2 is constrained in the extent of its activities by the fundamental laws of physics and thermodynamics particularly the laws that describe diffusion and molecular binding. Moreover, it could be speculated that the very earliest contact of this virus with the human mucosa defines the pathogenesis of this infection.

## Author contribution statement

Marko Popovic; Jennifer H. Martin; Richard J Head: Conceived and designed the experiments; Performed the experiments; Analyzed and interpreted the data; Contributed reagents, materials, analysis tools or data; Wrote the paper.

## Funding statement

This work was supported by the Ministry of Science, Technological Development and Innovation of the Republic of Serbia (Grant No. 451-03-47/2023-01/200026).

## Data availability statement

Data included in article/supp. material/referenced in article.

## Declaration of competing interest

The authors declare that they have no known competing financial interests or personal relationships that could have appeared to influence the work reported in this paper.
