# Supplementary material for: COVID infection in 4 steps: Thermodynamic considerations reveal how viral mucosal diffusion, target receptor affinity and furin cleavage act in concert to drive the nature and degree of infection in human COVID-19 disease
Source: Heliyon. 2023 Jun 12;9(6):e17174. doi: 10.1016/j.heliyon.2023.e17174 (PMC10259165; doi:10.1016/j.heliyon.2023.e17174)
Supplement: Multimedia component 1 [file mmc1.docx]

**9. SUPPLEMENTARY DATA
for the article “COVID infection in 4 steps: Thermodynamic considerations reveal how viral mucosal diffusion, target receptor affinity and furin cleavage act in concert to drive the nature and degree of infection in human COVID-19 disease.”**

9.1 Model description

Virus particles enter into upper airways with inhaled air and partly absorbed by the mucus surface. Those absorbed in the mucus diffuse through the mucus towards the cell surface. Once they reach the cell surface the virus particles bind to host cell receptors. On the other hand, the part of the viruses that was not absorbed by the mucus surface and remained in the air are carried by the air deeper into the airways. The goal of this analysis is to find the distribution of virus particles between the air, mucus surface, cell surface area and host cell receptors.

The process described above consists of three steps: viral adsorption onto the mucus surface, diffusion through the mucus and attachment to host cell receptors. The process can be modelled, and is displayed in a simplified form, in Figure S.1. Virus particles enter the upper airways dispersed in the inhaled air, designated with N. The inhaled air comes into contact with the mucus surface. A part of the virus particles is absorbed by the mucus surface, represented with A. The mucus thickness is *x*. Next, the viruses diffuse through the mucus and reach the membrane surface, B. State B is also known as the periciliary layer. Finally, the virus particles undergo the binding reaction and a part of them binds to host cell receptors, designated with C. Thus, the virus particles can be dispersed in air (N), free in the surface mucus (A), free in the mucus close to membrane surface (B) or bound to the membrane surface (C). The concentration of the virus in the air is *n*, the concentration of the virus in the mucus surface layer is *a*, the concentration of the free virus at the membrane surface is *b* and the concentration of the virus bound to the membrane surface is *c*. We are interested in how *n,* *a*, *b* and *c* change with time.


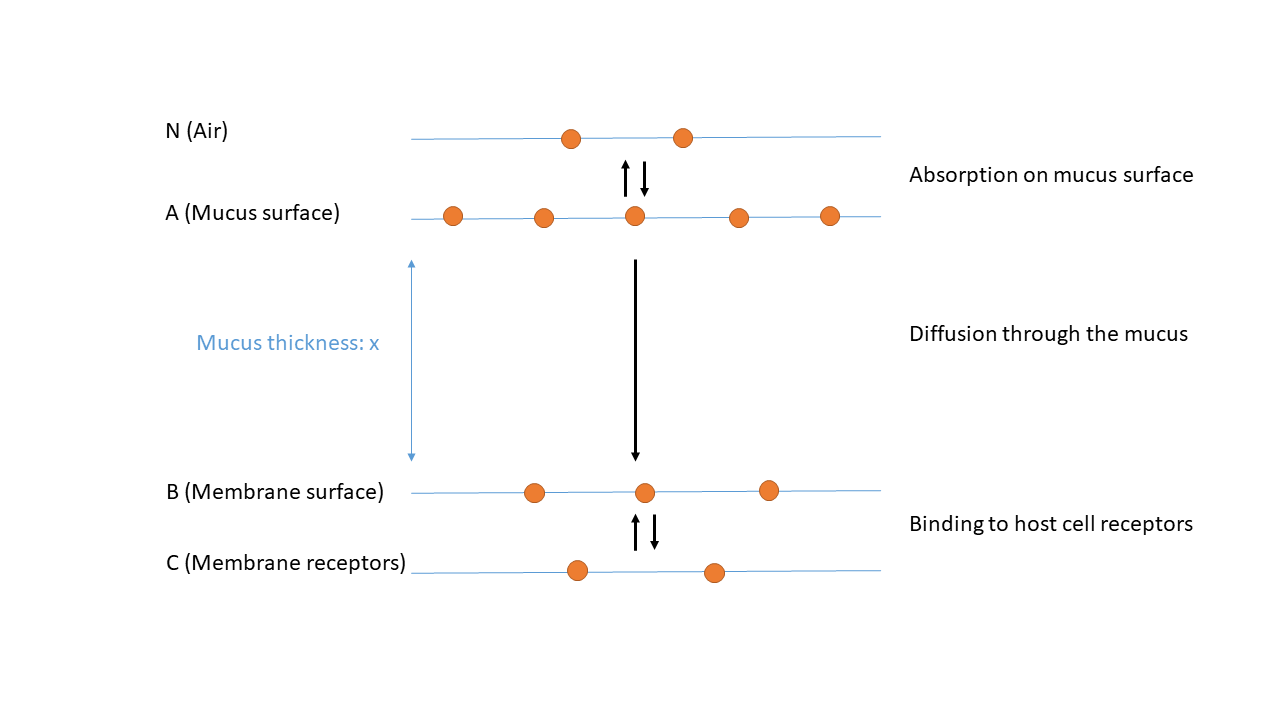


**Figure S.1:** Simplified representation of virus entry into mucus, diffusion through mucus and binding to host cell receptors. The orange circles represent virus particles that occupy the four available states: dispersed in air, suspended in mucus surface layer, free on membrane surface and bound to membrane receptors. The black double arrows (⇄) represent the absorption and binding processes, which are fast and are thus in pseudo-equilibrium. The black single arrow (→) represents the diffusion process, which is slow and is out of equilibrium.

To solve this problem, we will make four assumptions:

1. The diffusion process can be described by Fick’s law, which is applicable to most biological processes.
2. The diffusion process occurs at a lower rate than the absorption and binding process, and is thus the rate-limiting step. This means that the virus will take a lot of time to diffuse from the mucus surface (A) to the membrane surface (B). But, once at the surface, it will bind very fast. Similarly, the absorption process represents a partition of virus particles between nonpolar air and polar mucus. This process is also much faster than diffusion. Thus, the processes of absorption and binding can be assumed to be close to equilibrium. This assumption simplifies the mathematic treatment of the problem, but can be removed, at the expense of longer equations.
3. The concentration of host cell receptors (r) is much greater than the dissociation constant of the virus (K). This assumption is not problematic, since the dissociation constant is in the nanomolar range, while there are many host cells in the tissue. Again, the assumption can be removed if the equations are made a little longer.
4. Virus particles are polar and dissolve better in aqueous mucus solution than in air. Virus particles are normally assembled and dissolved in aqueous solutions, such as the cytoplasm, extracellular fluid, blood etc. Moreover, coronaviruses have an envelope consisted of a phospholipid bilayer, from which project the heads of polar spike proteins with carbohydrate extensions [Neuman and Buchmeier 2016; Neuman et al., 2011, 2006]. Thus, the surface of coronavirus particles and other viruses are highly polar and prefer polar environments over environments that are nonpolar. This means that virus particles will have a much greater solubility in mucus, which is a polar aqueous solution, than in nonpolar air. Again, the assumption can be removed, at the expense of longer equations.

With these three assumptions, the system of equations we are solving becomes

1. Distribution of virus particles between air and mucus surface, described by the partition coefficient, *f*.

$f=\frac{n}{a}$

where *n* and *a* are the concentrations of virus particles in the air and at the mucus surface, respectively.

1. Fick’s law of diffusion: 
   $J=-D\frac{a-b}{x}$
   where *J* is the flow rate of virus particles, *D* is the diffusion coefficient, *a*the concentration of virus particles at the mucus surface, *b* concentration of virus particles at the membrane surface and *x* mucus thickness [Atkins and de Paula, 2014, 2011].
2. Dissociation constant:

$K=\frac{b \cdot r}{c}$
where *r* is the receptor concentration and *c* the concentration of virus particles bound to the receptor [Popovic and Popovic, 2022; Du et al., 2016].

1. Conservation: 
   $m=a+b+c+n$
   where *m* is the total concentration of viruses taken into the organism.

The system of equations specified above is solved, using the four assumptions. The final result are the concentrations of virus particles in the four states, as a function of time.

$\frac{a}{m}=e^{-t/\tau}+\frac{K}{r}$

$\frac{b}{m}=\frac{K}{r}\left( 1-e^{-t/\tau} \right)$

$\frac{c}{m}=1-e^{-t/\tau}$

$\frac{n}{m}=f\left( e^{-t/\tau}+\frac{K}{r} \right)$

where τ is the time constant, specific for the system, defined by the diffusion coefficient and mucus thickness

$\tau=\frac{D}{x^{2}}$

This solution will be justified in the next section, where the solution procedure is shown.

9.2 Solution of the equations: Absorption, diffusion and binding of SARS-CoV and SARS-CoV2 variants.

- - 1. **The model and underlying equations in more detail**

The system we are considering is shown in Figure S.1. An initial inoculum first enters the airways with air, which is designated as state N. The concentration of virus particles in the air is represented with *n*. Some viruses are absorbed by the mucus surface, designated as state A. The concentration of viruses at the mucus surface is represented with *a*. Then, virus particles slowly start diffusing towards the cell membrane surface (State B). The concentration of unbound viruses at cell membrane surface is designated with *b*. The cell membrane surface is at a distance *x* from the mucus surface, which is the path that the virus particles must diffuse. Finally, once the viruses reach the surface, a part of them binds to the host cells (State C). The concentration of bound virus particles is designated with *c*. Thus, the system we are considering consists of an absorption process from N to A, a diffusion process from A to B, and a chemical reaction from B to C.

We are interested in finding how the concentrations of viruses, *n*, *a*, *b* and *c*, change with time. In the analysis, we will make three assumptions. First, the virus diffusion process is described by the Fick’s law. This assumption is not problematic, since most biological processes obey the Fick’s law. Second, the diffusion process is the rate limiting step. In other words, diffusion is much slower than the absorption and binding processes. The viruses will take a lot of time to reach the cell surface, but once there, they will bind very fast. Thus, the binding process will be at pseudo-equilibrium and we will not have to consider *k_on_* and *k_off_* rate constants. Similarly, distribution of virus particles between a polar mucus and nonpolar air environments is also a very fast process. This assumption should not be a problem either, since molecular binding, and distribution of particles between polar and nonpolar environments are in most cases very fast processes. Moreover, this assumption can be removed, although in that case the equations will be a little more complicated. Third, the receptor concentration, *r*, at the cell surface is much greater than the virus dissociation constant, *K*. Dissociation constants of most viruses in the nanomolar range. On the other hand, the receptor concentration should be higher than this as there are many cells in tissues. This assumption can also be removed, at the expense of longer equations. Finally, the fourth assumption states that virus particles are polar and prefer polar mucus environment over nonpolar air. This assumption is justified by the polar surface of coronaviruses, consisting of the polar heads of the envelope lipids, spike proteins and carbohydrates [Neuman and Buchmeier, 2016; Neuman et al., 2011, 2006].

Virus particles enter the airways, dispersed in air. Once the air comes into contact with the mucus surface. The mucus and air differ greatly in their physical properties. Air is gaseous and nonpolar, while the mucus is a polar aqueous solution. Thus, the virus particles that were all initially in air distribute between the nonpolar air and polar mucus. The distribution of virus particles is described by the partition coefficient, *f*

$f=\frac{n}{a}$ (S.1)

where *n* and *a* are the concentrations of virus particles in the air and at the mucus surface, respectively [Straathof, 2013]. This is the first starting equation.

The flow of virus particles from A to B is described by the Fick’s law [Atkins and de Paula, 2014, 2011]

$J=-D\frac{a-b}{x}$ (S.2)

Where *J* is the flow of virus particles, *D* is the diffusion coefficient, *a* is the concentration of viruses at the mucus surface, *b* the virus concentration at the cell membrane surface and *x* the thickness of the mucus layer (Figure S.1).

The virus particle flow *J* can easily be converted into rate at which particles leave state *A*, *R_a_*. Virus particle flow *J* is defined as

$J=\frac{1}{S}\frac{dN_{a}}{dt}$ (S.3)

Where *S* is the surface area of the membrane, *N_a_* the number of viruses on the mucus surface (layer A) and *t* time. In other words, *J* is the number of viruses that passes through a unit area per unit time. On the other hand, the rate at the virus concentration changes at the mucus surface is

$R_{a}=\frac{da}{dt}=\frac{d\left( {N_{a}}/V \right)}{dt}$ (S.4)

Which can be rearranged into

$R_{a}=\frac{1}{V}\frac{dN_{a}}{dt}$ (S.5)

Thus, *J* and *R_a_* are very similar quantities, differing only in use of surface area or volume, respectively. The ratio of surface area of the mucus layer to its volume is given in Figure S.2.

The top plane of the mucus surface, designated with *S*, is its top surface with a surface area *S*. The surface opposite to it is the cell membrane surface. These two surfaces are equal (*S*) and are separated by a mucus layer, with a thickness *x*. The volume between these two surfaces is simply the product of the surface area, *S*, and the distance between them, *x*.

$V=S\cdot x$ (S.6)

This means that the equation (4) can be rewritten as

$R_{a}=\frac{1}{S\cdot x}\frac{dN_{a}}{dt}=\frac{1}{x}\left( \frac{1}{S}\frac{dN_{a}}{dt} \right)=\frac{1}{x}\cdot J$ (S.7)

Substituting this in the Fick’s law (1) gives

$x\cdot R_{a}=-D\frac{a-b}{x}$ (S.8)

Which is combined with equation (3) to give

$x\cdot\frac{da}{dt}=-D\frac{a-b}{x}$ (S.9)

This is the form of the Fick’s law that will be used in the derivation and is the second starting equation.


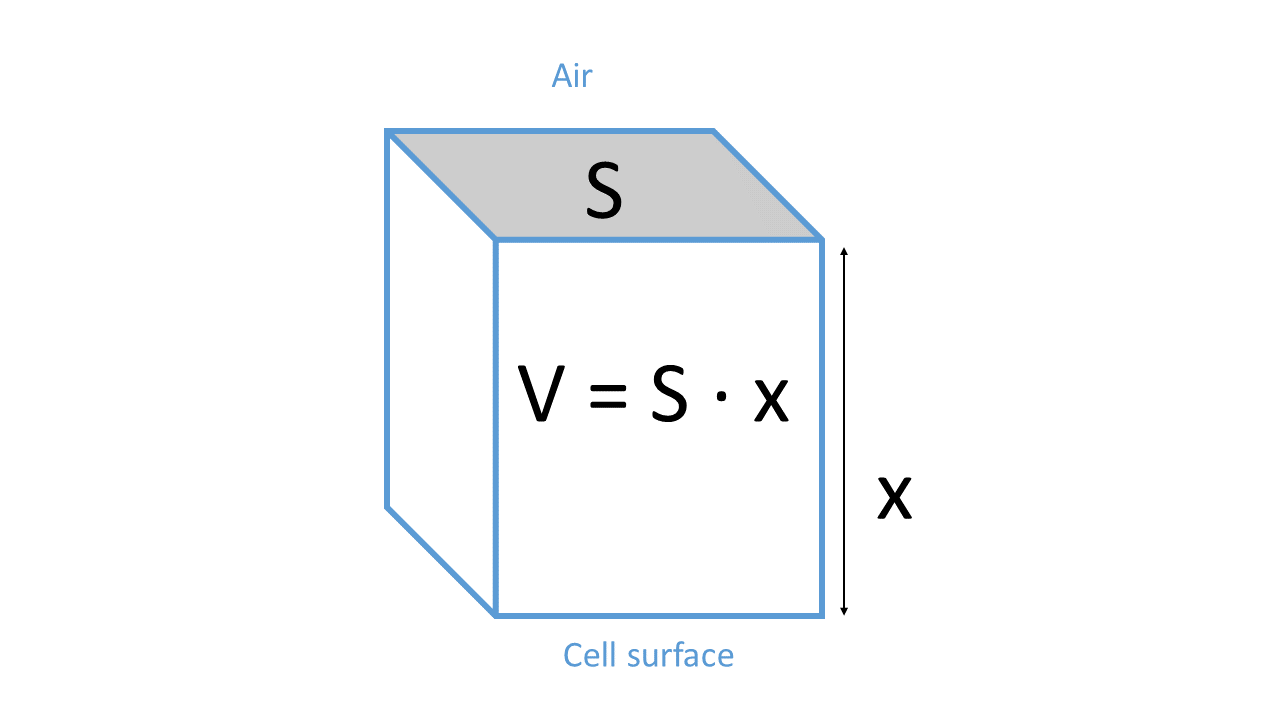


**Figure S.2:** The relationship of surface area and volume of the mucus layer. A fragment of the mucus layer is shown, which is located between the air and cell surface. It has a surface area *S*, thickness *x* and volume *V* = *S* ∙ *x*.

The third starting equation defines the equilibrium constant describing the binding process. The dissociation constant, *K*, relates the concentration of free virus particles at the cell membrane surface, *b*, concentration of host cell receptors, *r*, and concentration of virus particles bound to receptors, *c* [Popovic and Popovic, 2022; Du et al., 2016].

$K=\frac{b \cdot r}{c}$ (S.10)

Finally, the fourth equation is the conservation of matter. Virus particles can either be in the air, N, at the mucus surface, A, unbound at the cell membrane surface, B, or bound to the host cell receptor, C. Thus, the number of virus particles that entered, *m*, is divided into four parts

$m=a+b+c+n$ (S.11)

Where *n* is the concentration of virus particles in the air, *a* is the concentration of viruses at the mucus surface, *b* is the concentration of unbound viruses at the host cell membranes, and *c* is the concentration of viruses bound to the host membranes.

- - 1. *Concentration of free viruses at the mucus surface with time*

The equations (S.1), (S.9), (S.10) and (S.11) represent the starting point for the derivation: distribution, Fick’s law, dissociation constant and conservation. The goal is to find equations that describe the change in the virus concentrations *n*, *a*, *b* and *c* with time. This is a system of 4 equations with 4 unknowns and is solvable. The derivation will begin by substituting the distribution, binding constant and conservation equations into the Fick’s law. This will eliminate *n*, *b* and *c* and will leave *a* as the only unknown. The resulting equation will be solved for *a* as a function of time. Finally, the result for *a* will be substituted into the binding constant and conservation equations to find *b* and *c*, respectively.

First, the conservation equation (S.1) is rearranged to express *n* through *a*.

$n=a\cdot f$ (S.12)

Then, conservation equation (S.11) is first rearranged to express *c*, using *a*, *b* and *n*.

$c=m-a-b-n$ (S.13)

Then, *n* is eliminated by substituting equation (S.12), resulting in

$c=m-a-b-af$ (S.14a)

$c=m-b-a\left( 1+f \right)$ (S.14b)

The fourth assumption states that virus particles are polar and dissolve much better in polar mucus than in nonpolar air. This means that the concentration of virus particles in mucus is much greater than in air: *a* >> *n*. The partition coefficient *f* is defined as *f* = *n*/*a*, which means that the partition coefficient will be very small: *f* << 1. Thus, we can set (1 + *f*) ≈ 1 and equation (S.14b) reduces to

$c=m-b-a$ (S.15)

This equation is then substituted into the dissociation constant equation (9) to eliminate *c*.

$K=\frac{b \cdot r}{m-a-b}$ (S.16)

The resulting equation is then rearranged to express *b* through *a*.

$K\left( m-a-b \right)=b \cdot r$ (S.17a)

$K m-K a-K b=b r$ (S.17b)

$K m-K a=b r+K b$ (S.17c)

$K m-K a=b \left( r+K \right)$ (S.17d)

$b=\frac{K m-K a}{\left( r+K \right)}$ (S.17e)

$b=\frac{K \left( m- a \right)}{\left( r+K \right)}$ (S.17f)

Finally, equation (17f) can be simplified, using the assumption 3 that the receptor concentration is much greater than the dissociation constant: *r >> K*. This implies that *r + K* ≈ *r* and transforms equation (17f) into

$b=\frac{K}{r} \left( m- a \right)$ (S.18)

Equation (S.18) is now substituted into the Fick’s law equation (S.9), to eliminate *b*.

$x\cdot\frac{da}{dt}=-\frac{D}{x}\left[ a-\frac{K}{r} \left( m- a \right) \right]$ (S.19)

This equation can be rearranged to extract *r* in front of the parentheses.

$x\cdot\frac{da}{dt}=-\frac{D}{x}\frac{1}{r}\left[ ar-K \left( m- a \right) \right]$ (S.20)

The equation above is a simple differential equation, which can be solved by separating the variables d*a* and d*t* to different sides and integrating.

$\frac{da}{dt}=-\frac{D}{x^{2}}\frac{1}{r}\left[ ar-K \left( m- a \right) \right]$ (S.21a)

$\frac{da}{dt}=-\frac{D}{x^{2}}\frac{1}{r}\left[ ar-Km+Ka \right]$ (S.21b)

$r\frac{1}{\left[ ar-Km+Ka \right]}da=-\frac{D}{x^{2}}dt$ (S.21c)

$\int r\frac{1}{\left[ ar-Km+Ka \right]}da=-\int\frac{D}{x^{2}}dt$ (S.21d)

The equation is now integrated. The variable parameters are *a* and *t*, while *r*, *K*, *D* and *x* are constants.

$r\int\frac{1}{\left[ ar-Km+Ka \right]}da=-\frac{D}{x^{2}}\int dt$ (S.22a)

$r\int\frac{1}{\left[ ar-Km+Ka \right]}da=-\frac{D}{x^{2}}t+C'$ (S.22b)

*C’* represents the integration constant, which will be determined later. The solution of the integral on the left hand side is

$\int\frac{1}{\left[ ar-Km+Ka \right]}da=\frac{1}{r+K}\ln\left[ a\left( K+r \right)-Km \right]$ (S.23)

Substituting this into equation (S.22b) gives

$r\frac{1}{r+K}\ln\left[ a\left( K+r \right)-Km \right]=-\frac{D}{x^{2}}t+C'$ (S.24)

The last equation can be simplified, using the third assumption that *r* >> *K*, meaning that *r*+*K*≈*r.*

$r\frac{1}{r}\ln\left[ ar-Km \right]=-\frac{D}{x^{2}}t+C'$ (S.25a)

$\ln\left[ ar-Km \right]=-\frac{D}{x^{2}}t+C'$ (S.25b)

The equation above is almost the solution for part *a*. However, it contains the integration constant *C’.* The integration constant can be easily determined, using the initial conditions of our system. In the beginning of the process, at *t* ≈ 0, the virus particles have just arrived with the air. Since the process of absorption of virus particles from the air is fast, the air and mucus surface are in pseudo-equilibrium described by equation (S.1): *f* = *n*/*a*. This can be rearranged into *n* = *a f*. On the other hand, diffusion is a much slower process, meaning that no particles had time to diffuse to the host membranes and bind to the receptors. This means that the virus concentration at the host membrane and bound virus concentration are both zero: *b* = *c* = 0. Thus, the conservation equation (S.11) becomes:

$m=a+b+c+n$ (S.26a)

$m=a+0+0+af$ (S.26b)

$m=a\left( 1+f \right)$ (S.26c)

The fourth assumption states that virus particles dissolve much better in mucus than in air: *a* >> *n*. The ratio of solubilities in air and mucus define the partition coefficient *f* = *n* / *a*. Thus, the partition coefficient is very small *f* << 1, meaning that 1 + *f* ≈ 1. This is substituted into equation (S.26c) to give

$m\approx a$ (S.27)

In other words, most virus particles are located on the mucus surface, A.

Substituting this initial condition (*a*=*m* at *t*=0) into equation (S.25b) gives

$\ln\left[ mr-Km \right]=-\frac{D}{x^{2}}\cdot0+C'$ (S.28a)

$\ln\left[ m\left( r-K \right) \right]=C'$ (S.28b)

Again, the assumption that *r* >> K means that *r* – *K* ≈ *r*. This simplifies the equation above and gives us the value of the integration constant.

$C^{'}=\ln\left( mr \right)$ (S.29)

Equation (S.29) is now substituted into equation (S.25b) to remove the integration constant

$\ln\left[ ar-Km \right]=-\frac{D}{x^{2}}t+\ln\left( mr \right)$ (S.30)

The equation above is now solved for *a*. First the logarithms are removed, by raising both sides to the exponent.

$ar-Km=\exp\left[ -\frac{D}{x^{2}}t+\ln\left( mr \right) \right]$ (S.31)

Then, the exponent product rule is used: e*^p+q^*=e*^p^*∙e*^q^*.

$ar-Km=\exp\left[ -\frac{D}{x^{2}}t \right]\cdot\exp\left[ \ln\left( mr \right) \right]$ (S.32a)

$ar-Km=\exp\left[ -\frac{D}{x^{2}}t \right]\cdot mr$ (S.32b)

$ar-Km=mr\cdot e^{-\frac{D}{x^{2}}t}$ (S.32c)

The equation is now rearranged give the final result for *a*.

$ar=mr\cdot e^{-\frac{D}{x^{2}}t}+Km$ (S.33a)

$ar=m\left( r\cdot e^{-\frac{D}{x^{2}}t}+K \right)$ (S.33b)

$\frac{ar}{m}=r\cdot e^{-\frac{D}{x^{2}}t}+K$ (S.33c)

$\frac{a}{m}=\frac{1}{r}\left( r\cdot e^{-\frac{D}{x^{2}}t}+K \right)$ (S.33d)

$\frac{a}{m}=e^{-\frac{D}{x^{2}}t}+\frac{K}{r}$ (S.33e)

Equation above gives the fraction of viruses at the mucus surface, *a*/*m*, as a function of time. It can be simplified, by grouping the diffusion constant and mucus thickness into a new constant, *τ* = *D*/*x*².

$\frac{a}{m}=e^{-t/\tau}+\frac{K}{r}$ (S.34)

This represents the end of the *a*-part of the solution.

- - 1. *Concentration of free virus particles at the membrane surface with time*

The solution for the *b*-part is found by substituting the solution for *a*/*m* into the modified dissociation constant equation. Equation (S.18) is first rearranged.

$b=\frac{K}{r} \left( m- a \right)$ (S.35a)

$b=\frac{K}{r} m\left( 1- \frac{a}{m} \right)$ (S.35b)

$\frac{b}{m}=\frac{K}{r} \left( 1- \frac{a}{m} \right)$ (S.35c)

Next equation (28) is substituted into (29c), resulting in

$\frac{b}{m}=\frac{K}{r}\left[ 1- \left( e^{-t/\tau}+\frac{K}{r} \right) \right]$ (S.36)

This equation is now rearranged

$\frac{b}{m}=\frac{K}{r}\left[ 1- e^{-t/\tau}-\frac{K}{r} \right]$ (S.37a)

$\frac{b}{m}=\frac{K}{r}\left[ \left( 1- \frac{K}{r} \right)-e^{-t/\tau} \right]$ (S.37b)

Since *r* >> *K*, we can set (1 – *K*/*r*) ≈ 1. This simplifies the equation into

$\frac{b}{m}=\frac{K}{r}\left[ 1-e^{-t/\tau} \right]$ (S.38)

This is the final solution of the *b*-part of the problem.

- - 1. *Concentration of virus particles bound to the membrane*

The c-part of the problem is solved by substituting the solutions for the a-part and b-part into the conservation equation. We start from the conservation equation, which was previously modified into equation (S.15). We divide equation (S.15) with *m* to give

$c=m-a-b$ (S.39a)

$\frac{c}{m}=1-\frac{a}{m}-\frac{b}{m}$ (S.39b)

Then we substitute the equations (S.34) and (S.38)

$\frac{c}{m}=1-\left( e^{-t/\tau}+\frac{K}{r} \right)-\frac{K}{r}\left[ 1-e^{-t/\tau} \right]$ (S.40a)

$c=1-e^{-t/\tau}-\frac{K}{r}-\frac{K}{r}+\frac{K}{r}e^{-t/\tau}$ (S.40b)

$c=\left( 1-2\frac{K}{r} \right)-e^{-t/\tau}\left( 1-\frac{K}{r} \right)$ (S.40c)

Since *r* >> *K*, we can set (1 – *K*/*r*) ≈ 1 and (1 – 2*K*/*r*) ≈ 1. This simplifies the equation into

$c=1-e^{-t/\tau}$ (S.41)

This is the result for the c-part of the problem.

- - 1. *Concentration of virus particles in air*

The air above the mucus is in pseudo-equilibrium with the mucus surface. The pseudo-equilibrium state is described by the partition coefficient *f*, through equation (S.1). Equation (S.1) can be rearranged into

$n=f\cdot a$ (S.42)

This equation is divided with *m* to give.

$\frac{n}{m}=f\cdot\frac{a}{m}$ (S.43)

Then *a*/*m* is substituted by equation (S.34).

$\frac{n}{m}=f\left( e^{-t/\tau}+\frac{K}{r} \right)$ (S.44)

This is the final solution to the problem.

- - 1. *Summary*

In summary, viral diffusion through the mucus and attachment to host cell receptors was modeled as a combination of absorption, diffusion and chemical reaction. The absorption process was represented by the partition coefficient. The diffusion process was represented by Fick’s law. The chemical reaction was represented by the dissociation constant equation. The diffusion process was assumed to be slower than the absorption and chemical reaction. The final solution to the problem is:

$\frac{a}{m}=e^{-t/\tau}+\frac{K}{r}$ (S.45)

$\frac{b}{m}=\frac{K}{r}\left( 1-e^{-t/\tau} \right)$ (S.46)

$\frac{c}{m}=1-e^{-t/\tau}$ (S.47)

$\frac{n}{m}=f\left( e^{-t/\tau}+\frac{K}{r} \right)$ (S.48)

Where:

$\tau=\frac{D}{x^{2}}$ (S.49)

The function of *a*/*m* is shown in Figures S.3 and S.4 for SARS-CoV and SARS-CoV-2. For small values of *t*, the graphs for SARS-CoV and SARS-CoV-2 are very similar (Figure S.3). For large values of *t*, the two graphs diverge (Figure S.4). The *K* values for Figures S.3 and S.4 were taken from [Walls et al., 2020].

**
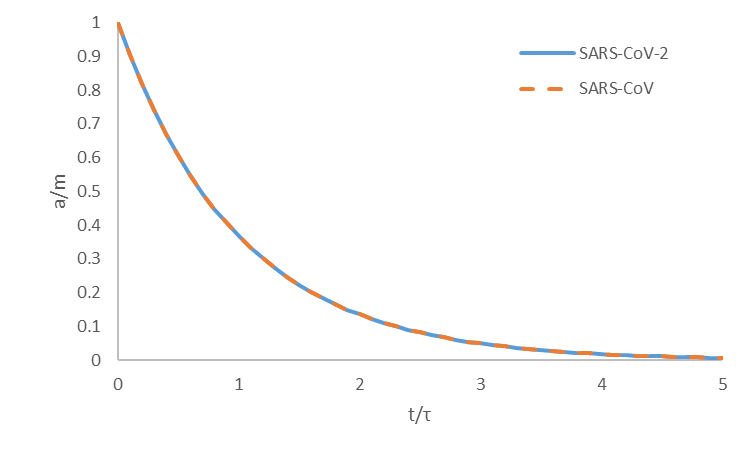
**

**Figure S.3:** Concentration of virus particles at the surface mucus as a function of time, for small time values. Since *t* is small, the exponent term in equation (S.45) dominates, which depends on virus diffusion properties summarized by *τ*. Since SARS-CoV and SARS-CoV-2 particles are of almost identical size and construction, their diffusion properties are very similar and the *a*/*m* graphs are almost identical for small *t*.

**
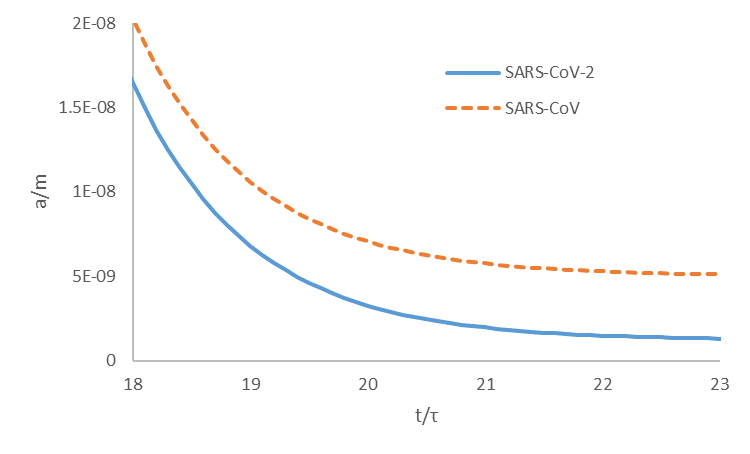
**

**Figure S.4:** Concentration of virus particles at the surface mucus as a function of time, for large time values. When *t* is large, the exponent term in equation (S.45) becomes very small, meaning that *a*/*m* is determined by the dissociation equilibrium constant, *K*. Since *K* is different for SARS-CoV and SARS-CoV-2, the graphs of *a*/*m* will be different at larger *t* values.

- 1. **Gibbs energy of binding and pathogenesis**

The formulas derived above can be used to explain the differences between clinical pictures of SARS-CoV and SARS-CoV-2 infections. The formula describing the change in virus concentration in inhaled air with time is

$\frac{n}{m}=f\left( e^{-t/\tau}+\frac{K}{r} \right)$ (S.49)

where *n* is the virus concentration in air, *m* the original amount of viruses that entered the organism, *f* the partition coefficient for viruses between air and mucus, *t* time, *τ* time constant (describing diffusion properties of virus particles), *K* dissociation constant for antigen-receptor binding and *r* receptor concentration on host cells.

A volume of air with viruses is taken into the airways through the nose, travelling from the nasal cavity to the alveoli. As it travels, it gives more and more viruses to the surrounding airway tissue. The more the air travels, the longer the time it has been in contact with the tissue, *t*. The air carrying viruses has taken some time to reach the lower airways, having to pass first through the nasal cavity, oral cavity, larynx, trachea etc. This means that for the lower airways *t* is large.

The large value of *t* greatly simplifies the function describing the virus concentration in air. This means that in the lower airways (where *t* → ∞) the equation simplifies into

$\frac{n}{m}=f\left( e^{-t/\tau}+\frac{K}{r} \right)$ (S.50a)

$\frac{n}{m}=f\left( e^{-\infty/\tau}+\frac{K}{r} \right)$ (S.50b)

$\frac{n}{m}=f\left( e^{-\infty}+\frac{K}{r} \right)$ (S.50c)

$\frac{n}{m}=f\left( 0+\frac{K}{r} \right)$ (S.50d)

Thus, the final equation describing the concentration of virus particles in the lower airways is

$\frac{n}{m}=\frac{f}{r} K$ (S.51)

The parameter *r* is the receptor (ACE2) concentration in the host tissue. Since SARS-CoV and SARS-CoV-2 viruses both attack respiratory pathways, the parameter *r* is the same for both viruses. The parameter *f* depends on the properties of the virus particle surface. Since SARS-CoV and SARS-CoV-2 have a very similar morphology [Neuman and Buchmeier, 2016; Neuman et al., 2011, 2006], their surfaces are very similar and the *f* parameter is essentially identical for the two viruses. Thus, the only parameter that differs between SARS-CoV and SARS-CoV-2 is the dissociation constant, *K*.

The equation above says that the concentration of viruses that arrives to lower airways is proportional to the dissociation constant K. We can use equation (S.51) to compare the concentrations of viruses that arrive to the lower airways. The parameters *r* and *f* are the same for both viruses. Thus, the ratio of concentrations of virus particles that reach the lower respiratory pathways depends only on the ratio of the dissociation constants

$\frac{n(SARS-CoV)}{n(SARS-CoV-2)}=\frac{K(SARS-CoV)}{K(SARS-CoV-2)}$ (S.52)

Where *n*(SARS-CoV) and *n*(SARS-CoV-2) are the concentrations of SARS-CoV and SARS-CoV-2 virus particles that reach the lower respiratory pathways, respectively, while *K*(SARS-CoV) and *K*(SARS-CoV-2) are the dissociation constants of the SARS-CoV and SARS-CoV-2, respectively.

The dissociation constant is proportional to the exponent of the standard Gibbs energy of binding, meaning that small changes in the latter will lead to great changes in the former. Dissociation constant, *K*, is related to the standard Gibbs energy of binding, Δ*_B_G⁰*, through the equation

$K=\exp\left( +\frac{\Delta_{B}G^{0}}{RT} \right)$ (S.53)

where *R* is the universal gas constant and *T* temperature (the sign before Δ*_B_G⁰* is plus instead of minus, since: Δ_B_G⁰ = - *RT* ln (*K_B_*) and *K_B_* = 1/*K*, where *K_B_* is the binding constant, the reciprocal of the dissociation constant) [Popovic and Popovic, 2022]. Equation (S.53) shows that the dissociation constant *K* is proportional to the exponent of the standard Gibbs energy of binding, Δ*_B_G⁰*. This means that small changes in Δ*_B_G⁰* will make great changes in *K* and thereby in the concentration of viruses in the lower airways, through equation (S.52).

In summary, the consideration above means that if a virus A has a more negative (smaller) standard Gibbs energy of binding than virus B, the dissociation constant of virus A will be much smaller than that of virus B, due to the exponent in equation (S.53). This means that the concentration of virus A particles in the lower respiratory pathways will be much smaller than that of virus B, according to equation (S.52). If more virus B particles reach the lower respiratory pathways, virus B will infect lower respiratory pathways more often than virus A. This will now be tested with SARS-CoV and SARS-CoV-2 viruses.

- 1. **Comparison of SARS-CoV and SARS-CoV-2**

According to equations (S.52) and (S.53), the ratio of concentrations of SARS-CoV and SARS-CoV-2 viruses reaching the lower airways depends on standard Gibbs energies of binding of the two viruses. Dissociation constants of SARS-CoV and SARS-CoV-2 (Hu-1 variant) were reported by Walls et al. [2020]. The dissociation constant, *K*, of SARS-CoV is 5.0 nM, while that of SARS-CoV-2 is 1.2 nM, at 30°C [Walls et al., 2020]. These values can be substituted into equation (S.53), to find standard Gibbs energy of binding, Δ*_B_G⁰*. This results in Δ*_B_G⁰* = -48.2 kJ/mol for SARS-CoV and Δ*_B_G⁰* = -51.8 kJ/mol for SARS-CoV-2. Thus, Δ*_B_G⁰* of SARS-CoV is only a little less negative (greater) than that of SARS-CoV-2.

The *K* values of the two viruses can be used to find the ratio of concentrations reaching the lower airways, using equation (S.52).

$\frac{n(SARS-CoV)}{n(SARS-CoV-2)}=\frac{K(SARS-CoV)}{K(SARS-CoV-2)}$ (S.54a)

$\frac{n(SARS-CoV)}{n(SARS-CoV-2)}=\frac{5.0 nM}{1.2 nM}$ (S.54b)

$\frac{n(SARS-CoV)}{n(SARS-CoV-2)}=4.2$ (S.54c)

This means that the concentration of SARS-CoV reaching the lower airways is 4.2 times greater than that of SARS-CoV-2. Therefore, even though SARS-CoV has just a slightly less negative (greater) standard Gibbs energy of binding than SARS-CoV-2, much more SARS-CoV particles will reach the lower airways.

**Supplementary material references**

Atkins, P. and de Paula, J. (2014). *Physical Chemistry: Thermodynamics, Structure, and Change, 10^th^ ed.,* New York: W.H. Freeman and Company.

Atkins, P. and de Paula, J. (2011). *Physical Chemistry for the Life Sciences, 2^nd^ ed.,* New York: W.H. Freeman and Company.

Du, X., Li, Y., Xia, Y.-L., Ai, S.-M., Liang, J., Sang, P., Ji, X.-L., et al. (2016). Insights into Protein–Ligand Interactions: Mechanisms, Models, and Methods. *International Journal of Molecular Sciences*, *17*(2), 144. MDPI AG. Retrieved from <http://dx.doi.org/10.3390/ijms17020144>

Neuman, B.W. and Buchmeier, M.J. (2016). Supramolecular architecture of the coronavirus particle. *Advances in Virus Research*, 96, 1-27. <https://doi.org/10.1016/bs.aivir.2016.08.005>

Neuman, B. W., Kiss, G., Kunding, A. H., Bhella, D., Baksh, M. F., Connelly, S., Droese, B., Klaus, J. P., Makino, S., Sawicki, S. G., Siddell, S. G., Stamou, D. G., Wilson, I. A., Kuhn, P., & Buchmeier, M. J. (2011). A structural analysis of M protein in coronavirus assembly and morphology. *Journal of structural biology*, *174*(1), 11–22. <https://doi.org/10.1016/j.jsb.2010.11.021>

Neuman, B. W., Adair, B. D., Yoshioka, C., Quispe, J. D., Orca, G., Kuhn, P., Milligan, R. A., Yeager, M., & Buchmeier, M. J. (2006). Supramolecular architecture of severe acute respiratory syndrome coronavirus revealed by electron cryomicroscopy. *Journal of virology*, *80*(16), 7918–7928. <https://doi.org/10.1128/JVI.00645-06>

Popovic, M. and Popovic, M. (2022). Strain Wars: Competitive interactions between SARS-CoV-2 strains are explained by Gibbs energy of antigen-receptor binding. *Microbial Risk Analysis*. <https://doi.org/10.1016/j.mran.2022.100202>

Straathof, A.J.J. (2013). Thermodynamics in multiphase biocatalysis. In Urs von Stockar, ed., *Biothermodynamics: The Role of Thermodynamics in Biochemical Engineering*, Lausanne: EPFL Press, pp. 289-314.

Walls, A. C., Park, Y. J., Tortorici, M. A., Wall, A., McGuire, A. T., & Veesler, D. (2020). Structure, Function, and Antigenicity of the SARS-CoV-2 Spike Glycoprotein. *Cell*, *181*(2), 281–292.e6. <https://doi.org/10.1016/j.cell.2020.02.058>
